# An All-vanadium Continuous-flow Photoelectrochemical Cell for Extending State-of-charge in Solar Energy Storage

**DOI:** 10.1038/s41598-017-00585-y

**Published:** 2017-04-04

**Authors:** Zi Wei, Yi Shen, Dong Liu, Fuqiang Liu

**Affiliations:** 1 Department of Mechanical Engineering, University of Massachusetts Lowell, Lowell, MA 01854 USA; 2 Department of Materials Science and Engineering, University of Texas at Arlington, Arlington, TX 76019 USA

## Abstract

Greater levels of solar energy storage provide an effective solution to the inherent nature of intermittency, and can substantially improve reliability, availability, and quality of the renewable energy source. Here we demonstrated an all-vanadium (all-V) continuous-flow photoelectrochemical storage cell (PESC) to achieve efficient and high-capacity storage of solar energy, through improving both photocurrent and photocharging depth. It was discovered that forced convective flow of electrolytes greatly enhanced the photocurrent by 5 times comparing to that with stagnant electrolytes. Electrochemical impedance spectroscopy (EIS) study revealed a great reduction of charge transfer resistance with forced convective flow of electrolytes as a result of better mass transport at U-turns of the tortuous serpentine flow channel of the cell. Taking advantage of the improved photocurrent and diminished charge transfer resistance, the all-V continuous-flow PESC was capable of producing ~20% gain in state of charge (SOC) under AM1.5 illumination for ca. 1.7 hours without any external bias. This gain of SOC was surprisingly three times more than that with stagnant electrolytes during a 25-hour period of photocharge.

## Introduction

Large-scale storage of intermittent solar energy and subsequent conversion to electricity potentially offer a sustainable solution to global energy shortage, yet is subject to availability of efficient process. Storing solar energy in H_2_ via light-driven water splitting has been regarded as a promising path^1^; however, it suffers from a low conversion efficiency and poor storage option due to gaseous H_2_. To circumvent these issues, tremendous amount of efforts have been devoted to alternative processes to improve conversion efficiency^2, 3^ and develop better storage options^4^. Besides, several pioneer works integrating energy storage systems such as supercapacitors^5^, lithium-ion batteries^6, 7^, lithium–iodine redox batteries^8, 9^, and photoelectrochemical (PEC) conversion^10^ have shown great promise for convenient storing solar energy; however, none of these systems have been particularly successful because of a multitude of problems such as low energy density, complex cell construction involving both lithium metal and liquid electrolyte, and insufficient photovoltage which requires an external power supply to charge the systems.

Recent research has demonstrated a unique all-vanadium (all-V) PEC storage cell (PESC) in which redox-couple reactants remain in aqueous solutions during both photocharge and discharge. Utilizing one-electron PEC reactions of vanadium redox species^11^, similar to those in a vanadium redox flow battery (VRB), such system possesses all merits inherent to VRBs: fast electrochemical kinetics, excellent efficiencies, decoupled and scalable power and energy density, long cycle life, and reasonable storage volumes. It has been shown that the all-V PESC achieved a high Faradaic efficiency of 95%^12^ and peak incident-photon-to-current efficiency (IPCE) of 45% in solar energy storage^13^. In addition, nanostructured TiO_2_ photocatalysts^14^, WO_3_/TiO_2_ hybrid photoelectrodes to render additional electron storage capability^11, 15, 16^, and alternative supporting acid^13^ (*i.e*., methanesulfonic acid) have been studied in the all-V PESC to further improve the performance. However, despite these efforts, the fundamental challenge for the all-V PESC is the loss of driving potential due to concentration polarization as a result of liquid-phase ionic and species transport resistance, which limits the photocurrent and photocharging depth. So far only a 6% gain of state of charge (SOC) over a 25-hour period of photocharge was achieved^12^. Provided that kinetically-fast redox species have been employed in the system, the key to improving conversion efficiency and extending SOC in solar energy storage is to design an effective continuous-flow cell that utilizes forced convective transport of the reactants throughout the cell as much as possible, such that diffusive transport is only required over short dimensions. Consequently, consumed vanadium species during PEC reactions can be immediately replenished to achieve a deeper depth of photocharge. Although the fundamental impact of flow on fuel cells, redox flow batteries, and other PEC devices^17–19^ has been documented, the mass transport of reactants in deeply-charged flow PEC solar storage cells has been rarely studied.

In this work, we report an all-V continuous-flow PESC to attain significant improvement in photocharging depth. The impact of forced convective flow on cell performance such as photocurrent, solar energy conversion, electrochemical as well as PEC reactions has been investigated. In addition, 3D numerical simulation has been conducted to gain more insights into the cell operation. Compared to those using stagnant electrolytes, the continuous-flow PESC shows great advantages in enhancing the photocurrent and remarkably 3.3 times improvement in photocharging depth.

## Results and Discussion

### Assembly of the all-V continuous-flow PESC cell

**Figure 1:**
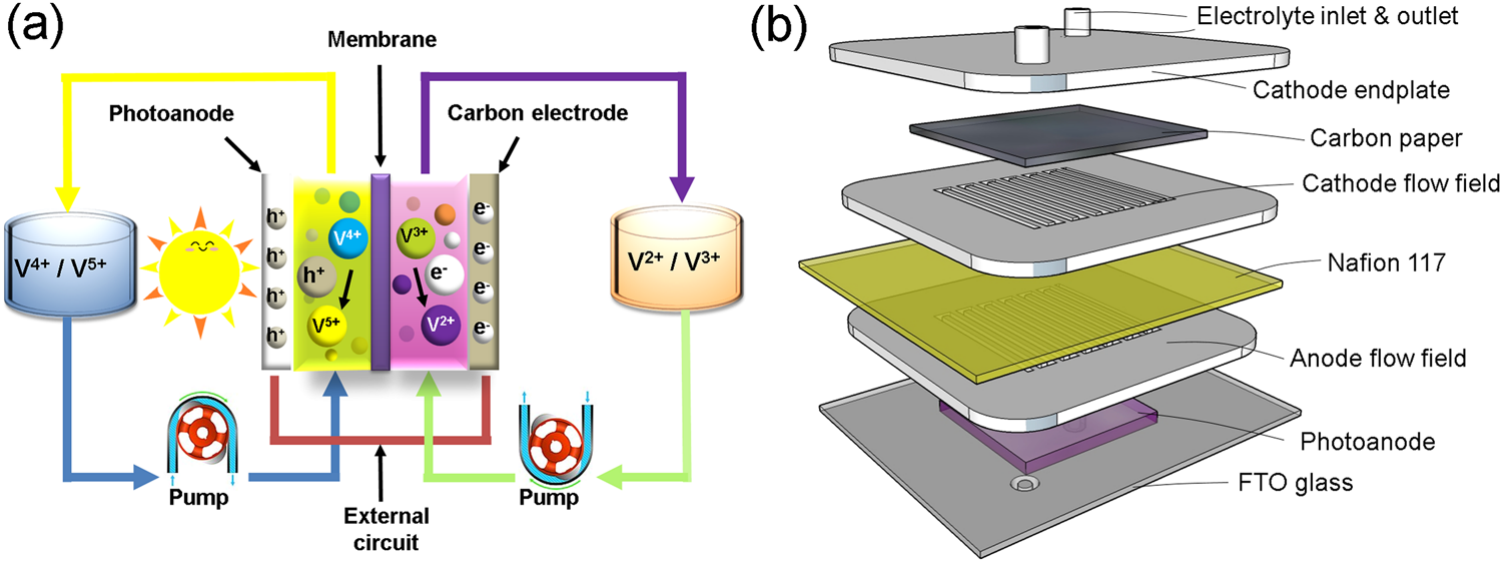
(**a**) Schematic representation of the all-V continuous-flow PESC setup, including the cell, pumps, and electrolyte storage tanks. (**b**) Exploded view of the all-V continuous-flow storage cell. V^4+^ and V^5+^ denote vanadium species of VO^2+^ and VO_2_^+^, respectively. During operation, two different vanadium redox electrolytes, i.e., VO^2+^ and V^3+^ (balanced with 3 M H_2_SO_4_ and separated by an ion-conducting membrane), are used as the anolyte (in contact with the photoanode) and catholyte (in contact with a carbon-paper cathode), respectively. Under photocharge, reactions at the photoanode and cathode are VO^2+^ + H_2_O → VO_2_^+^ + e^−^ + 2H^+^ and V^3+^ + e^−^ → V^2+^, respectively.

Schematic diagrams of the all-V continuous-flow PESC, experimental setup, and detailed cell construction (active area of 1 in × 1 in) are presented in Fig. 1. A Nafion 117 membrane was used to separate two different vanadium electrolytes, i.e., 0.01 M VO^2+^ and 0.01 M V^3+^ (balanced with 3 M H_2_SO_4_) in the anode and cathode compartments, respectively. Two acrylic (Virtualplast, Israel) serpentine flow fields, machined by an Epilog Helix 24 laser cutter, were attached to both side of the Nafion 117 membrane. The depth and width of the flow channel are 1 mm and 1 mm, respectively. A TiO_2_ photoanode coated on a FTO glass was placed at the anode compartment, while a carbon paper (SGL, AA30) serving as the counter electrode was inserted between the cathode flow field and endplate. The layers of the cell were glued together to prevent leaking.

### Effect of vanadium redox flow on PEC response

**Figure 2:**
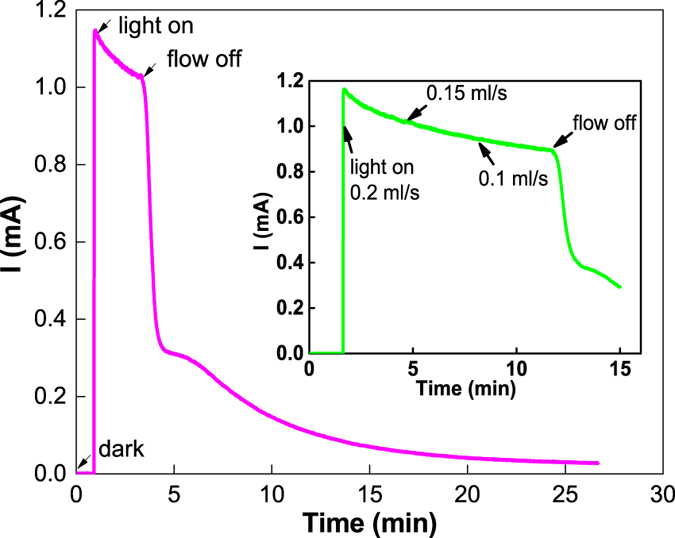
Photoelectrochemical response of the all-V continuous-flow PESC to flow of vanadium electrolytes. The anolyte and catholyte are 0.01 M VO^2+^ and 0.01 M V^3+^ (balanced with 3 M H_2_SO_4_), respectively. The photoanode is a TiO_2_ electrode. The flow rates of the anolyte and catholyte were varied simultaneously.

The unbiased photocharging of the all-V continuous-flow PESC and its thermodynamic requirement have been demonstrated in our previous work^12^. Figure 2 presents the impact of electrolyte flow rate on photocurrent produced from the cell under AM1.5. Two sets of experiments were conducted. In the 1^st^ set of test, both the anolyte and catholyte were fed into the cell at a flow rate of 0.2 ml/s. Upon AM1.5 illumination at 1 min, the photocurrent, resulting from two half-cell reactions of VO^2+^ + H_2_O → VO_2_^+^ + e^−^ + 2H^+^ and V^3+^ + e^−^ → V^2+^, promptly jumped to 1.15 mA and then decreased as a result of the well-known anodic overshoot (AO)^17^. After 3 min, complete shutting off the flow caused the photocurrent quickly drop to 0.33 mA, followed by a gradual decay to 0.03 mA at 25 min when the vanadium species in the flow fields were depleted. It is evident that the forced convective flow of electrolytes plays a critical role in the PEC reactions, resulting in at least 5-fold improvement in photocurrent. Note that the all-V continuous-flow PESC is essentially a photocharged VRB, which could take full advantage of fast kinetics of vanadium redox under convective flow.

In the 2^nd^ set of experiment (inset of Fig. 2), after light was switched on at 2 min, the photocurrent rapidly increased as a spike, which closely resembles that in the 1^st^ set of experiment, then gradually leveled off. The initial electrolyte flow rate was 0.2 ml/s, and then reduced to 0.15 ml/s and 0.1 ml/s at 5 min and 8 min, respectively. The PEC performance of the cell, although largely affected by the forced convective flow as seen in the 1^st^ set of test, was imperceptibly impacted by reducing flow rate from 0.2 to 0.1 ml/s. This is different to what is routinely observed in VRBs where electrolyte flow rate is a crucial factor and cell limiting current densities increase continuously with flow rate as a result of enhanced mass transport^20^. In this study, however, the cell photocurrent is determined by a complex interplay of multiple factors, including PEC processes at the photoanode, electrochemical reactions at the cathode, and transport of redox species in the electrolytes. Our results seem to indicate that under forced convective flow of electrolytes in the studied range of flow rates, the cell performance is solely dependent on the fundamental photo/electrochemical reaction rate at the electrode/electrolyte interfaces.

The observed photocurrents in Fig. 2, in contrast to our previous studies^12, 14^ using similar vanadium redox and photoelectrode but stagnant electrolytes, are more than 5 times higher. This exceptional enhancement is largely attributed to the fast reaction kinetics of vanadium species that quickly scavenge the photogenerated charges with minimized recombination: holes tend to react with VO^2+^ at the photoanode while electrons reduce V^3+^ at the cathode. Most important, introduction of forced convective flow in the all-V continuous-flow PESC is believed to not only reduce mass transport polarization^21^, but also expedite the abovementioned surface processes. It is known that electron-hole recombination consumes a large fraction of the photogenerated charge carriers, taking a nanosecond time scale at room temperature^22, 23^, for wide-bandgap semiconductors such as TiO_2_^24^. Typically, under illumination charges could be trapped by the surface states of TiO_2_, and the induced recombination is responsible for rapid decrease of photocurrent^25^. Especially, when reactive redox species near the photoanode surface have been depleted by PEC reactions, charges could be accumulated at the TiO_2_ surface, further deteriorating the PEC performance. This phenomenon is supposed to be largely eliminated by the continuous-flow cell design, since the depleted vanadium redox could be effectively replenished.

**Figure 3:**
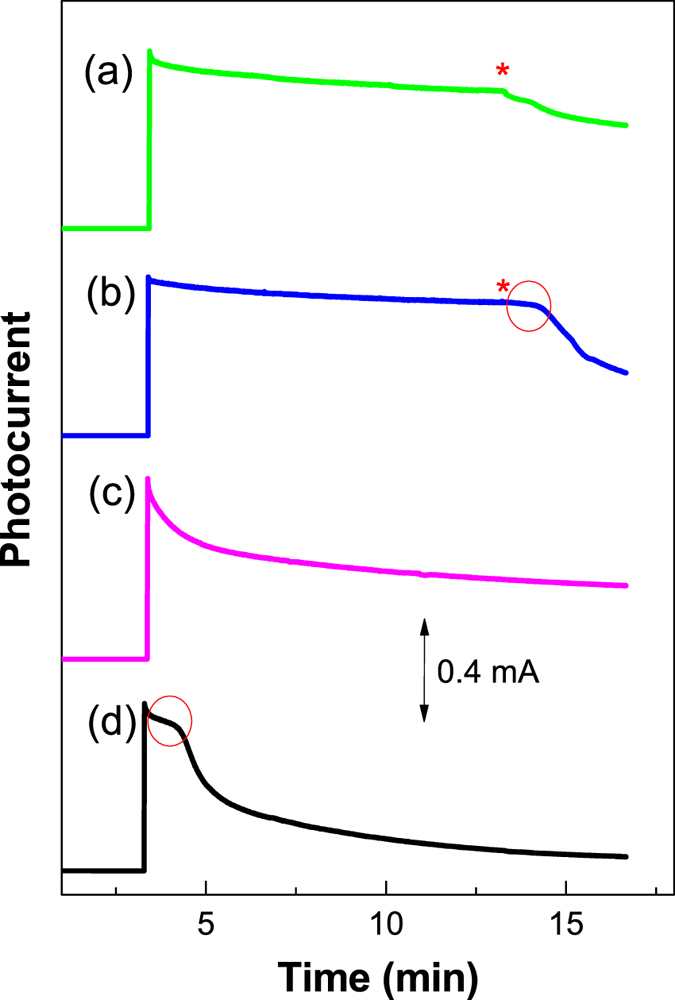
Photoelectrochemical response of the all-V continuous-flow PESC to different electrolyte flow conditions. Both the anode and cathode flow (at 0.2 ml/s) was on at the beginning of the tests, and light was turned on at 3 min. (**a**) At 13 min the anode flow was switched off (denoted by an asterisk). (**b**) At 13 min the cathode flow was switched off. (**c**) Anode flow was switched off at 3 min. (**d**) Cathode flow was switched off at 3 min.

To further investigate the impact of flow on cell performance, different flow conditions for the photoanode and cathode were investigated. In both Fig. 3a and b, constant electrolyte flow rate at 0.2 ml/s was applied at the beginning for both sides of the cell. Light was switched on at 3 min, which produced fairly stable photocurrents. At 13 min either the anode flow (Fig. 3a) or cathode flow (Fig. 3b) was shut off (denoted by an asterisk) while the other side remained unchanged. Apparent decrease of photocurrent was observed for both cases, indicating that mass transport in both the anolyte and catholyte is important in the overall PEC performance. However, careful examination of the curves suggests drastically different photoresponse of the two cases. Upon shutting off the anode flow (Fig. 3a), the photocurrent immediately dropped, but gradually approached a plateau. In contrast, turning off the cathode flow resulted in a 1-min photocurrent shoulder (denoted by a red circle in Fig. 3b) followed by a more abrupt decay than that in Fig. 3a.

To distinguish the contribution of the anolyte and catholyte flow to the cell performance, asymmetric flow conditions with flow on only one side of the cell were adopted: stagnant anolyte and 0.2 ml/s of the catholyte (Fig. 3c); and 0.2 ml/s of the anolyte and stagnant catholyte (Fig. 3d). Comparing the two cases, once again, clearly suggests an important role of catholyte flow on the cell photoresponse. Interestingly, a similar 1 min photocurrent shoulder (denoted by a red circle in Fig. 3d) emerged after turning off the cathode flow, clearly resembling that in Fig. 3b.

### Electrochemical impedance spectroscopy study

**Figure 4:**
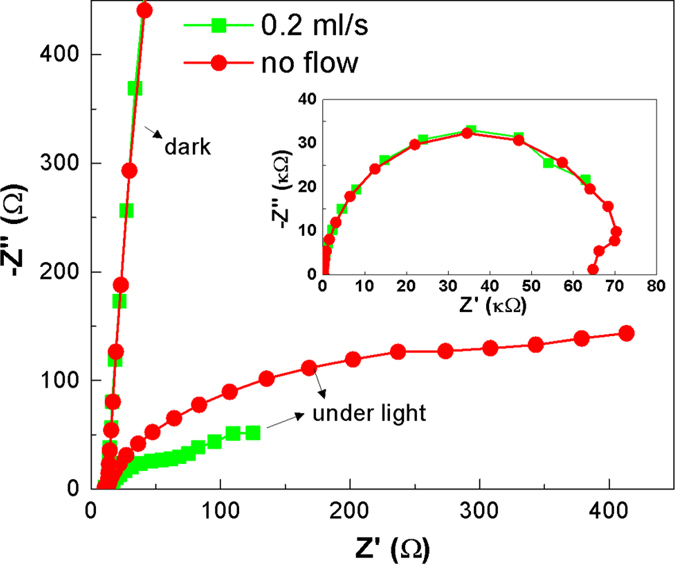
EIS spectra of the all-V continuous-flow PESC under dark/illumination and different flow conditions using 0.01 M vanadium redox species. The inset shows the full spectra under dark.

In order to understand the impact of electrolyte flow on kinetic charge-transfer processes in the cell, electrochemical impedance spectroscopy (EIS) was used to study the all-V continuous-flow PESC. The Nyquist plots in Fig. 4 under dark at different flow rates essentially overlap indicating negligible influence of electrolyte flow on electrochemical performance. The obtained large diameter (inset of Fig. 4) of the EIS spectra suggests extremely high charge transfer resistance. Upon AM1.5 illumination, especially under forced convective flow of electrolytes, the cell shows characteristic semicircles that represents multiple electrochemical interfaces for charge transport in the electrochemical system^26^. Under illumination but with no flow, the EIS curve bends toward the real axis in comparison to that under dark, suggesting a reduced charge transfer resistance. The low-frequency tail (a small portion with Z_re_ > 300 Ω) is related to Warburg mass transport due to sluggish diffusion of vanadium redox species. Upon introduction of flow, the overall Nyquist plot drastically shrinks and two clear semicircles emerge in high (Z_re_ < 70 Ω) and intermediate frequency (Z_re_ > 75 Ω) ranges, corresponding to the charge transfer processes at the carbon paper/V^3+^ and TiO_2_/VO^2+^ interfaces^13^, respectively. This suggests that both the anode and cathode are co-dominating the overall cell performance.

### Computational study

**Figure 5:**
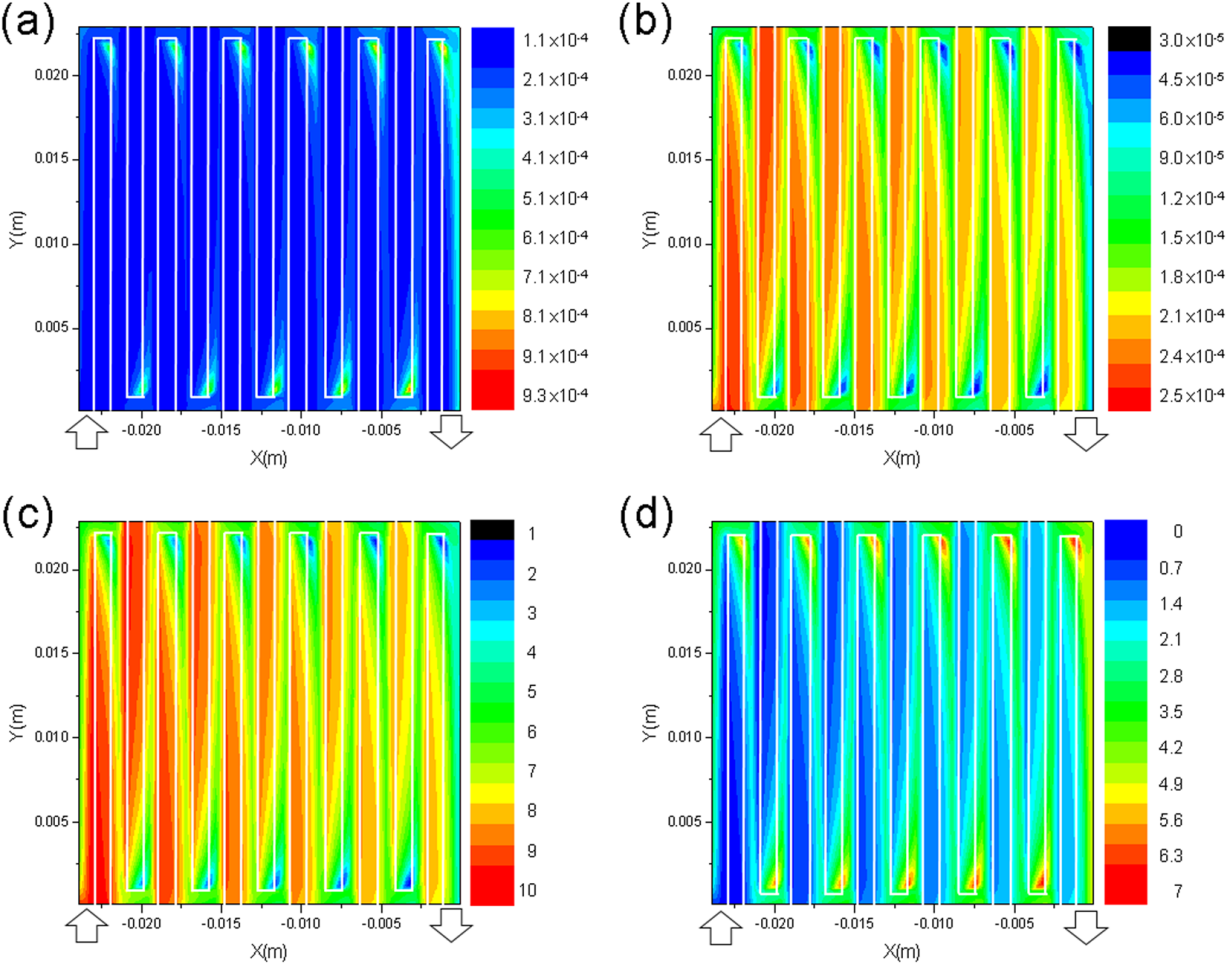
Simulated 2D concentration contour plots (in mole/m^3^) of four active species: (**a**) holes, (**b**) electrons, (**c**) VO^2+^, and (**d**) VO_2_^+^, along the midplane of the photoelectrode under a photocurrent of 0.5 mA and electrolyte flow rate of 0.01 ml/s.

A 3D multi-component PEC model, in consideration of momentum, charge generation and recombination, and PEC reactions, was developed for the photoanode half-cell (see Supplementary Information for details) to better understand the convection-enhanced PEC reactions. Figure 5 shows the 2D species distribution, including electrons, photons, VO^2+^, and VO_2_^+^, in the midplane of the TiO_2_ photoelectrode (Supplementary Information, Fig. S1) under a flow rate of 0.01 ml/s (VO^2+^ at 0.01 M) and a prescribed photocurrent of 0.5 mA. As expected, VO_2_^+^ redox species (Fig. 5d), generated by PEC oxidation of VO^2+^, increase its concentration along flow direction in the serpentine channel (the inlet and outlet are indicated on each plot), which is consistent with the VO^2+^ concentration contour plot in Fig. 5c. On the other hand, distribution of hole (Fig. 5a) and electron (Fig. 5b) concentration in the photoelectrode seems to be closely dependent on that of vanadium redox species. In the conducted steady-state simulation, the area with lower VO^2+^ concentration consumes less holes, which results in a relatively higher hole concentration in the photoelectrode. This will subsequently determine the electron concentration distribution in Fig. 5b because of charge recombination, which is proportional to both electron and hole concentrations (see the governing equations for electrons and holes in Supplementary Information). Besides, Fig. 5, where the serpentine flow field is superimposed on top of each figure, clearly shows the drastic impact of flow on species distribution and PEC reaction rate. The area in the photoelectrode immediately after each U-turn of the serpentine flow field appears to achieve the highest reaction rate. In Fig. 5c and d, the tortuous serpentine flow channel provides better convection at the U-turns and, hence, results in better mass transport of vanadium redox species and consequently higher reaction rates in the photoelectrode even under the land area. However, a higher flow rate of vanadium electrolyte, e.g., at 2 ml/s (Supplementary Information, Fig. S3), renders relatively uniform concentration distribution of different species, reasonably due to a larger supply of VO^2+^ to the cell.

**Figure 6:**
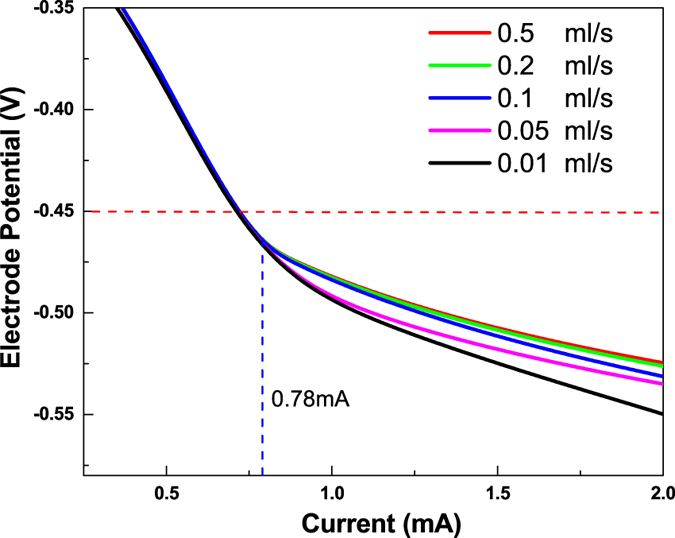
Simulated cathode electrode potential as a function of the current for an all-V continuous-flow PESC under different flow rates of V^3+^ (0.01 M). The estimated Fermi level (electrochemical potential) is indicated in the figure as a dashed line.

Complementary to the above studies, steady-state simulation was also performed on the cathode half-cell (see Supplementary Information for details of the model) to further unfold the impact of electrolyte flow. Electrochemical reduction reaction of V^3+^ + e^−^ → V^2+^ and its related transport processes were simulated at various applied electrode potentials *E*, which is defined as $E=\eta +{E}_{{V}_{3}-{V}_{2}}$ with *η* and ${E}_{{V}_{3}-{V}_{2}}$ representing the cathode overpotential (as the driving force for reduction reaction of V^3+^) and equilibrium potential of V^3+^/V^2+^, respectively. In Fig. 6 the electrode potential is plotted against the current under different flow rates of the catholyte (0.01 M V^3+^) from 0.01 to 0.5 ml/s. The results show that the impact of flow rate is trivial when the current is less than 0.78 mA, owing to large stoichiometric coefficients of the supplied V^3+^ ranging from 618 0.5 ml/s to 12 at 0.01 ml/s. These large stoichiometric coefficients also explain why the polarization curves in Fig. 6 do not show limiting currents. The impact of catholyte flow rate starts to emerge at higher currents because a substantially negative electrode potential is required, particularly, for small flow rates of V^3+^. However, experimental operation of the all-V continuous-flow PESC would not possibly reach the high-current conditions corresponding to *E* < −0.45 V (below the horizontal dash line in Fig. 6). Virtually, −0.45 V is the estimated Fermi level (*E*_*F*_) of TiO_2_ in the electrochemical scale, which is dependent upon the semiconductor’s surface properties^27, 28^. Essentially, the TiO_2_ Fermi energy sets the maximum driving potential for V^3+^ reduction at short circuit, i.e., ${E}_{F}-{E}_{{V}_{3}-{V}_{2}}$, under which the simulation results in Fig. 6 suggest no observable difference of current under a wide range of flow rates. This result is consistent with our experimental study that the PEC performance of the cell does not seem to be affected by the catholyte flow rate within the studied range.

The experimental and simulation findings have revealed a quizzical dependence of cell photoresponse on electrolyte flow. In good agreement with the observation in VRBs^20^, we discovered that the anolyte and catholyte flow impacts cell performance differently. In the all-V continuous-flow PESC, the 1-min shoulders in Figs 2 and 3b,d actually represent a two-stage decay of photocurrent, which is closely related to mass transport process of vanadium redox species in the cathode flow field. Though root cause of the abovementioned observation remains ambiguous and requires a profound investigation to clarify the impact of flow rates on cell performance^29–31^, several reasons are plausible. First of all, diffusivity of V^3+^ is about half that of VO^2+^ ^21^. Slow diffusive transport of V^3+^ to the carbon paper cathode could lead to a sizable loss of photocurrent, unless forced convection is employed. Second, overpotential at the cathode as the driving force for V^3+^ reduction is small. As discussed in our previous study^12^, the driving potential for the cathode reaction is only 0.24 V compared to 1.8 V at the photoanode under standard conditions. Therefore, depletion of V^3+^ in the cathode flow channels due to PEC reactions could create concentration polarization and shift the Nernst redox potential, thus causing an abrupt reduction of photoresponse after the observed photocurrent shoulder in Fig. 3. Albeit exhibiting excellent electrochemical kinetics, the V^3+^ reduction reaction is expected to be more dependent on vanadium redox concentration and requires convective flow to replenish V^3+^ supply and achieve higher current. This also suggests that the overall performance of the continuous-flow PEC cell seems to be dominated by the cathodic reaction. Further experimental study of the photoanode PEC performance using a H-shape electrochemical cell with stagnant electrolytes (Fig. S4) shows that AM1.5 illumination not only reduces the photoelectrode potential compared to that under dark, but also significantly enhances its limiting photocurrent that is much higher than that theoretically produced from the cathode as shown in Fig. 6. Additional light on and off cycles (Fig. S5), and linear sweep voltammograms (Fig. S6) are shown in the Supplementary Information.

### Long-term Photocharge

**Figure 7:**
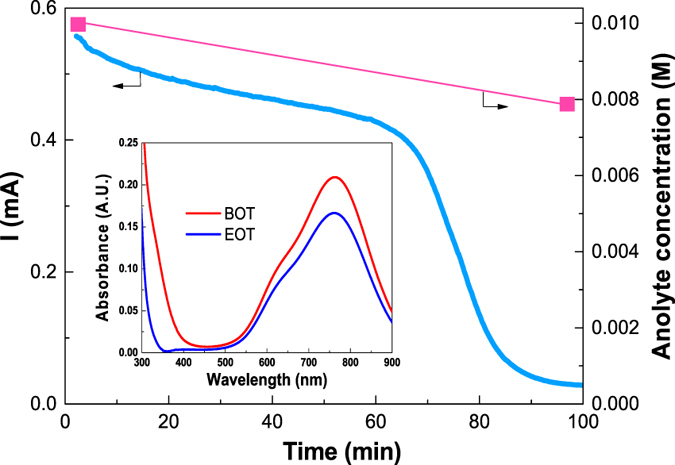
Continuous photocharging of the all-V continuous-flow PESC under AM 1.5 illumination using 0.01 M vanadium redox electrolytes. UV-Vis absorbance spectra of the BOT and EOT anolyte were shown in the inset. The BOT and EOT denote begin-of-test and end-of-test, respectively.

A continuous photocharge of the cell under AM1.5 illumination was conducted for 100 min to evaluate its long-term PEC characteristics. In Fig. 7 the photocurrent of the cell continuously declined during the entire testing window. Note that the beginning photocurrent is lower than what have been shown in Figs 2 and 3 because this test was conducted immediately after those. Approximately at 60 min, a sharp decrease of the photocurrent occurred mainly due to reduction of vanadium concentration that could shift the Nernst redox potential and therefore drastically diminish the driving electromotive force for photocharge. Besides, concentration loss and therefore the resultant overpotential particularly at the cathode could also contribute to the photocurrent decay. Photoelectrode corrosion and decomposition of the electrolyte might also contribute to the sharp drop of photocurrent, but are rather unlikely because of the proved stability of TiO_2_ and demonstrated high Faradaic efficiency^12^. To precisely assess the vanadium concentration change, UV-Vis absorption spectroscopy was utilized to analyze the anolyte. The inset of Fig. 7 shows the absorbance spectra of the beginning-of-test (BOT) and end-of-test (EOT) anolyte. According to Eq. [3], the EOT anolyte concentration is estimated to be 0.0079 M, suggesting that 21% of the vanadium redox species have been converted during this photocharging test.

### Faradaic and Solar-chemical-electricity Efficiencies

After investigating the all-V continuous-flow PESC and understanding impact of forced convective electrolyte flow, we can evaluate the cell Faradaic efficiency (η_F_) during the photocharge. According to Eq. [1], η_F_ is calculated to be 95%, which is attributed to fast reaction kinetics of vanadium redox and therefore suppressed H_2_/O_2_ evolution reactions^32^. The overall solar-chemical-electricity (SCE) conversion efficiency during the first hour of the photocharging test in Fig. 7 is estimated to be 0.6% assuming that the energy conversion efficiency of a VRB is 90% (Eq. [2]). This efficiency seems low, mainly due to the wide bandgap TiO_2_; however, to the best of our knowledge, it doubles the best solar-hydrogen-electricity efficiency using single state-of-the-art semiconductor material. The best unbiased solar-to-hydrogen efficiency using a single-junction PEC/PV device is ~1%, which yields an efficiency of only ~ 0.3% in conversion to electricity using a fuel cell with a typical 30% efficiency.

### Depth of Photocharge

Though a seemingly shallow charge depth (~21% change in SOC), estimated by VO^2+^ concentration change, was achieved during the 100-min photocharge, the present study actually represents a significant leap forward over our previous report^12^, where only a 6% change of SOC over a 25-hour period was demonstrated. This extended photocharging depth in solar energy storage is due to the applied forced convective flow in the all-V continuous-flow PESC, which effectively improves the photocurrent and allows for efficient conversion of vanadium redox even at fairly low concentration. Future research will focus on further improving charge depth of the cell by optimizing the flow channel and using nanostructured semiconductor materials.

## Conclusion

This study demonstrated the advantages of an all-V continuous-flow PESC for extending charging depth in PEC solar energy storage. This cell uses convective flow of electrolytes not only to prevent reactant depletion, but also to enhance interfacial reaction rates. This cell also takes advantage of high-kinetics vanadium redox species to reduce charge recombination and depress side reactions. With no external bias, the photocurrent produced by the cell was enhanced by 5 times in comparison to the conventional stagnant cell, mainly due to enhanced mass transport by the forced convective flow. Using a 3D CFD-based model, the U-turns of the tortuous serpentine flow channel of the cell were found to render the highest PEC reaction rate. This is further confirmed by the EIS study that forced convective flow of electrolytes helps greatly reduce charge transfer resistance in both sides of the cell. During photocharge, more than 3-fold increase of depth of charge (21% SOC) during 100-min was obtained with 95% faradaic efficiency in contrast to only 6% SOC gain demonstrated in our previous study during a 25-hour test.

## Experimental Methods

### Fabrication of photoelectrodes

Fluorine-doped tin oxide (FTO) glass substrates (2 in × 2 in) were prepared and cleaned by sonication in acetone (99.7%, Fisher Scientific, USA) for 20 min followed by methanol (99.8%, Fisher Scientific, USA) for 20 min, and then DI water. 0.5 g Degussa P25 (Evonik) TiO_2_ nanoparticles were mixed with 2.15 g α-terpineol (Fisher Scientific, USA), and then sonicated for 20 min. The obtained slurries were coated on the FTO glass substrates using a doctor blade to form a uniform film (active area of 1 in × 1 in) and then dried in an oven at 80 °C for 2 hour. The as-prepared photoanodes were sintered with air flow in a tube furnace at 500 °C for 1 hour^12, 14, 33^.

### PEC studies and characterization of the all-V continuous-flow PESC

The PEC characteristics of the all-V continuous-flow PESC were studied using a PARSTAT 2273 potentiostat. During operation of the cell, the VO^2+^ anolyte and V^3+^ catholyte in 10 ml storage tanks were continuously pumped to the cell (Fig. 1a), where the photoanode and carbon paper served as the working electrode (WE) and cathode, respectively. The photocurrent, as a result of the light-driven PEC reactions (the energy diagram is shown in Fig. S7), was measured using the protocol of zero-resistance ammetry (ZRA) with no externally applied bias. Solar irradiance was provided by an ozone-free solar simulator system (Newport, USA) paired with an AM1.5 global filter (Newport, USA). Electrochemical impedance spectroscopy (EIS) study was performed by applying an AC voltage of 10 mV to the cell in a frequency range from 10 mHz to 2 MHz.

The faradaic efficiency (η_F_) is calculated by the equation:

1 $${{\rm{\eta }}}_{F}=\frac{F{\rm{\Delta }}n}{{\rm{\Delta }}Q}$$

where Δn is the amount of reacted vanadium redox species during the test, F is the Faraday constant (96 485 C mol^−1^), and ΔQ is the generated charge. The overall solar-chemical-electricity conversion efficiency could be calculated according to

2 $${{\rm{\eta }}}_{overall}=\frac{{{\rm{\eta }}}_{dis}\times {{\rm{I}}}_{ph}\times {E}^{0}}{{P}_{in}}$$

where I_*ph*_ is the photocurrent, *E* ^0^ is the reversible potential of the redox couple (1.25 V), *P*_*in*_ is the incident solar power, and η_*dis*_ is the discharge efficiency which is estimated to be 90%.

To determine the concentration of vanadium redox species during operation, a small amount of electrolyte was extracted and analyzed in a quartz cuvette by a UV-vis spectrophotometer (PerkinElmer Lambda 35). A linear relationship between the peak (at 765 nm) absorbance (A) of vanadium redox and its concentration is assumed, according to the Beer–Lambert law, i.e.,

3 $${\rm{A}}=\varepsilon lc$$

where *ε* is the molar absorptivity, *l* is the path length of the cuvette, and *c* is the concentration of the targeted vanadium redox species.

### Computational studies

In order to theoretically study the influence of electrolyte flow on photoresponse of the all-V continuous-flow PESC, 3D multicomponent simulation was conducted using the SIMPLER algorithm in a commercial CFD software Fluent 6.3.26. User defined functions (UDF) were written to account for the transport and photo/electrochemical reactions of different species including vanadium redox ions, electrons, and holes. The mathematical models and details of the simulation are presented in the Supplementary Information.

## Electronic supplementary material

Supplementary information
