# Supplementary material for: An All-vanadium Continuous-flow Photoelectrochemical Cell for Extending State-of-charge in Solar Energy Storage
Source: Sci Rep. 2017 Apr 4;7:629. doi: 10.1038/s41598-017-00585-y (PMC5428687; doi:10.1038/s41598-017-00585-y)
Supplement: Supplementary file 1 — Supplementary information [file 41598_2017_585_MOESM1_ESM.pdf]

Supplementary information

An All-vanadium Continuous-flow Photoelectrochemical Cell for Extending State-of-charge in Solar Energy Storage

*Zi Wei, Yi Shen, Dong Liu, and Fuqiang Liu**

Email: fuqiang_liu@uml.edu

1. Numerical simulation

Numerical simulation has been widely employed to study photoelectrochemical water splitting[1,2] as well as vanadium flow batteries (VRBs)[3,4]. In the present study of the all vanadium (all-V) continuous-flow photoelectrochemical storage cell (PESC), the numerical models in the above studies have been adopted to investigate the impact of forced convective flow on performance of the storage cell.

1.1. 3D Steady-state simulation of the photoanode in the all-V Continuous-flow PESC

a. Model assumptions

The model is based on the following assumptions:

1) The electrolyte flow is incompressible and laminar.
2) All elements are isothermal.
3) Hydrogen and oxygen evolution reactions are neglected.
4) The dilute solution approximation is adopted for species transport.
5) The electrolyte density is constant and electrode properties are homogeneous.

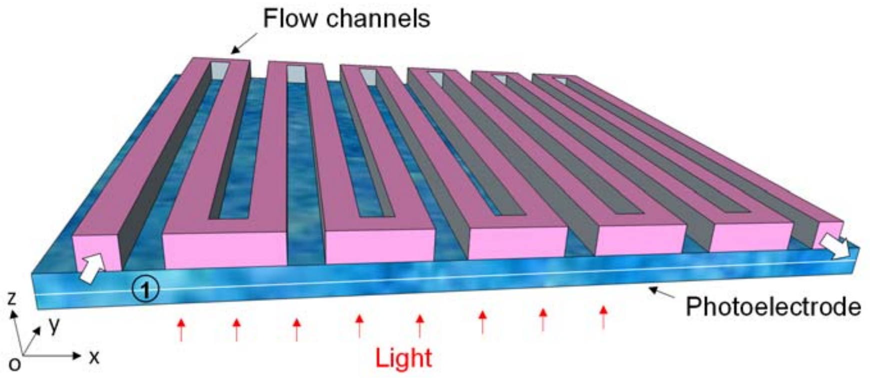

Figure S1. 3D computational domain of the photoanode of the all-V Continuous-flow PESC (thickness of the photoelectrode not in scale). The symbol ① indicates the midplane in the photoelectrode where 2D concentration contour plots of different species were studied (**Fig. 5** of the main text).

b. *Electrochemical reactions and transport characteristics*

Photons with energy higher than the TiO_2 bandgap could generate electron-hole pairs. The photogenerated holes (h^+) oxidize VO^{2+} according to the following reaction:

$$h^+ + VO^{2+} + H_2O \rightarrow VO_2^+ + 2H^+$$

The kinetics of the above light-driven reaction can be described by

$$j_1 = Fk_1 C_h{}^a C_{V^{4+}}{}^b$$

where j_1 is the current density, F is the Faraday's constant, k_1 is the standard rate constant for the photoelectrode, and $C_{V^{4+}}$ and C_h are the concentrations of VO^{2+} and hole, respectively. In this study, first-order reaction kinetics are assumed; therefore, both *a* and *b* are taken as unity.

c. *Governing equations and source terms*

Steady-state simulation was performed on the 3D domain in **Fig. S1**. The governing equations incorporating the aforementioned electrochemical reaction and the source terms accounting for the species generation/consumption are:

<u>Continuity:</u> $\nabla \cdot \vec{u} = 0$

<u>Momentum:</u> $\frac{1}{\varepsilon^2}\nabla \cdot (\rho \vec{u}\vec{u}) = -\nabla p - \frac{\mu}{K}\vec{u}$

where ρ is the density, ε is the porosity, μ is the electrolyte viscosity, p is pressure, and K is the permeability.

<u>Electrons</u> (only in the photoelectrode)**:** $-\nabla \cdot (D_e \nabla C_e) = \frac{\eta_{inj}\alpha(\lambda) I_0 e^{-\alpha(\lambda)z}}{N_A} - k_{recomb} C_e C_h$

where D_e , C_e , η_{inj} , $\alpha(\lambda)$, I_0 , z , and k_{recomb} are electron diffusion coefficient, electron concentration, electron injection efficiency, wavelength-dependent absorption coefficient, incident photon flux, distance to the semiconductor/electrolyte surface (see **Fig. S1**), and the charge recombination rate constant, respectively. N_A is the Avogadro's number. The 1st and 2nd terms on the RHS stand for the electron generation rate and consumption rate (due to charge recombination), respectively.

<u>Holes</u> (only in the photoelectrode)**:** $-\nabla \cdot (D_h \nabla C_h) = \frac{\eta_{inj}\alpha(\lambda) I_0 e^{-\alpha(\lambda)z}}{N_A} - k_{recomb} C_e C_h - a_1 \frac{j_1}{F}$

where a_1 is the specific active surface area of the photoelectrode (m^2/m^3). The 1st, 2nd, and 3rd terms on the RHS of the above equation represent the electron generation rate, consumption rate due to charge recombination, and consumption due to photoelectrochemical reaction with VO^{2+}, respectively.

Vanadium VO^2+^: $\nabla \cdot (\vec{u} C_{V^{4+}}) - \nabla \cdot (D_{V^{4+}}^{eff} \nabla C_{V^{4+}}) = S_{V^{4+}}$

where $S_{V^{4+}} = -a_1 \frac{j_1}{F}$ only in the photoelectrode. The effective diffusion coefficients of vanadium species in the porous electrodes are calculated using the following equation, i.e.,

$$D_i^{eff} = D_i \, \varepsilon^{1.5} \, .$$

Vanadium VO_2^+: $\nabla \cdot (\vec{u} C_{V^{5+}}) - \nabla \cdot (D_{V^{5+}}^{eff} \nabla C_{V^{5+}}) = S_{V^{5+}}$

where $S_{V^{5+}} = a_1 \frac{j_1}{F}$ only in the photoelectrode.

d. Boundary conditions

The simulation was performed under the steady-state condition with a constant current flowing into the photoelectrode (at $z = 0$ in **Fig. S1**). Non-flux wall boundary conditions are applied to all other surfaces. Vanadium redox concentrations at the inlet boundary are constant and velocity varies during the simulation. The simulation was conducted by the SIMPLER algorithm in a commercial CFD software Fluent 6.3.26. User defined functions (UDFs) were written to account for diffusivity and source terms for different species in the photoelectrode and flow channels. Some of the parameters employed in the simulation are listed in **Table S1**.

Table S1. Parametric properties used in the simulation [4-6]

Parameters	Value
Standard reaction rate constant, k_1	1.0×10^{-5}
Standard reaction rate constant, k_2	1.25×10^{-7}
Electron injection efficiency, η_{inj}	0.93
incident photon flux, I_0 (#/m^2/s)	2.8×10^{21}
Photoanode absorption coefficient, $\alpha@375nm$ (m)	300
Charge recombination rate constant, k_{recomb}	4.8×10^{7}
Porosity of the carbon paper, ε_c	0.78
Porosity of the photoanode, ε_{PE}	0.66
Permeability of the photoanode, K_{PE} (m^2)	1.0×10^{-11}
Permeability of the carbon paper, K_c (m^2)	5.0×10^{-11}
Specific surface area of photoanode, a_1 (m^{-1})	125000
Specific surface area of carbon paper, a_2 (m^{-1})	12645
Inlet concentration of V^{2+}, $C^0_{V^{2+}}$ (mole/l)	1.0×10^{-7}
Inlet concentration of V^{3+}, $C^0_{V^{3+}}$ (mole/l)	0.01
Inlet concentration of VO^{2+}, $C^0_{V^{4+}}$ (mole/l)	0.01
Inlet concentration of VO_2^{+}, $C^0_{V^{5+}}$ (mole/l)	1.0×10^{-7}
Active area, (in x in)	1.0 x 1.0
Channel dimension, depth x width (mm x mm)	1.0 x 1.0
Diffusion coefficient of V^{2+}, $D_{V^{2+}}$ (m^2/s)	2.4×10^{-10}
Diffusion coefficient of V^{3+}, $D_{V^{3+}}$ (m^2/s)	2.4×10^{-10}
Diffusion coefficient of VO^{2+}, $D_{V^{4+}}$ (m^2/s)	3.9×10^{-10}
Diffusion coefficient of VO_2^{+}, $D_{V^{5+}}$ (m^2/s)	3.9×10^{-10}

1.2. 3D Steady-state simulation of the cathode in the all-V Continuous-flow PESC

a. Electrochemical reactions

The electrochemical redox reaction occurring in the cathode half-cell is as follows:

$$V^{3+} + e^{-} \rightarrow V^{2+}$$

The transfer current j_2 for the above reaction is described as [7]:

$$j_2 = a_2 F k_2 \left(c_{V^{2+}} c_{V^{3+}}\right)^{0.5} \left[\exp\left(\frac{F}{2RT}\left(E - E_{V_3 - V_2}\right)\right) - \exp\left(-\frac{F}{2RT}\left(E - E_{V_3 - V_2}\right)\right)\right]$$

where a_2 is the specific active surface area of the carbon paper (m^2/m^3), E is the electrode potential, $E_{V_3-V_2}$ is equilibrium potential of V^{3+}/V^{2+} which is $E_{V_3-V_2} = \frac{RT}{F}\ln(C_{V^{3+}} / C_{V^{2+}}) - 0.255$, and k_2 is the standard rate constants for the cathode reaction. It should be noted that species migration is ignored here[8], because its contribution to species transport is not significant in the redox flow battery according to reference[4].

b. Governing equations and source terms

Simulation was conducted on the 3D domain in **Fig. S2**. The governing equations for flow, vanadium redox species, and electrode potential are listed in **Table S2**.

Table S2. Governing equations for the transport and electrochemical model

	Equations	Source Terms
Continuity	$\nabla \cdot \vec{u} = 0$	—
Momentum	$\frac{1}{\varepsilon^2}\nabla \cdot (\rho \vec{u}\vec{u}) = -\nabla p + S_u$	$S_u = -\frac{\mu}{K}\vec{u}$
V^{2+}	$\vec{u}\nabla C_{V^{2+}} - D_{V^{2+}}^{eff}\nabla^2 C_{V^{2+}} = S_{V^{2+}}$	$S_{V^{2+}} = -\frac{j_2}{F}$ in electrode
V^{3+}	$\vec{u}\nabla C_{V^{3+}} - D_{V^{3+}}^{eff}\nabla^2 C_{V^{3+}} = S_{V^{3+}}$	$S_{V^{3+}} = \frac{j_2}{F}$ in electrode
Electrode potential	$-\sigma_s^{eff}\nabla^2 E = S_s$	$S_s = -j_2$ in electrode

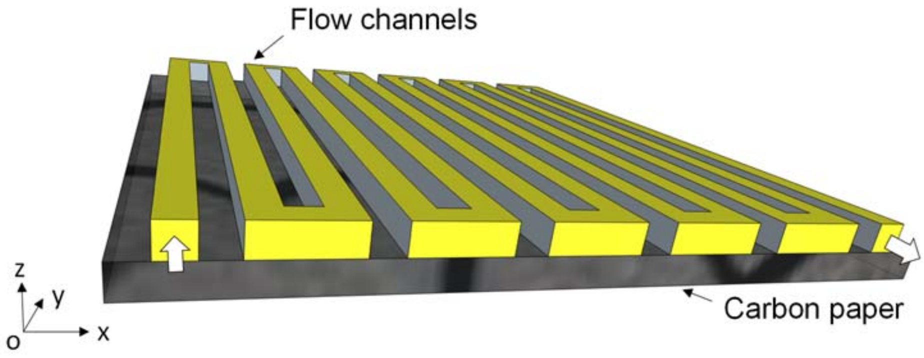

Figure S2. 3D computational domain of the cathode in the all-V Continuous-flow PESC (thickness of the carbon paper not in scale).

c. Boundary conditions

Steady-state simulation was performed under galvanostatic operation and a constant current density is applied to the carbon paper/current collector interface, i.e., $z = 0$ (**Fig. S2**)

$$-\sigma_s^{eff}\frac{\partial E}{\partial x} = -I$$

where E is the electrode potential. Non-flux wall boundary conditions are applied to all other surfaces. Vanadium redox concentrations at the inlet boundary remain constant and velocity varies during the simulation. The simulation was conducted at different current and the resultant electrode potential E at the carbon paper/current collector interface was plotted against the applied current in **Fig. 6** of the main text.

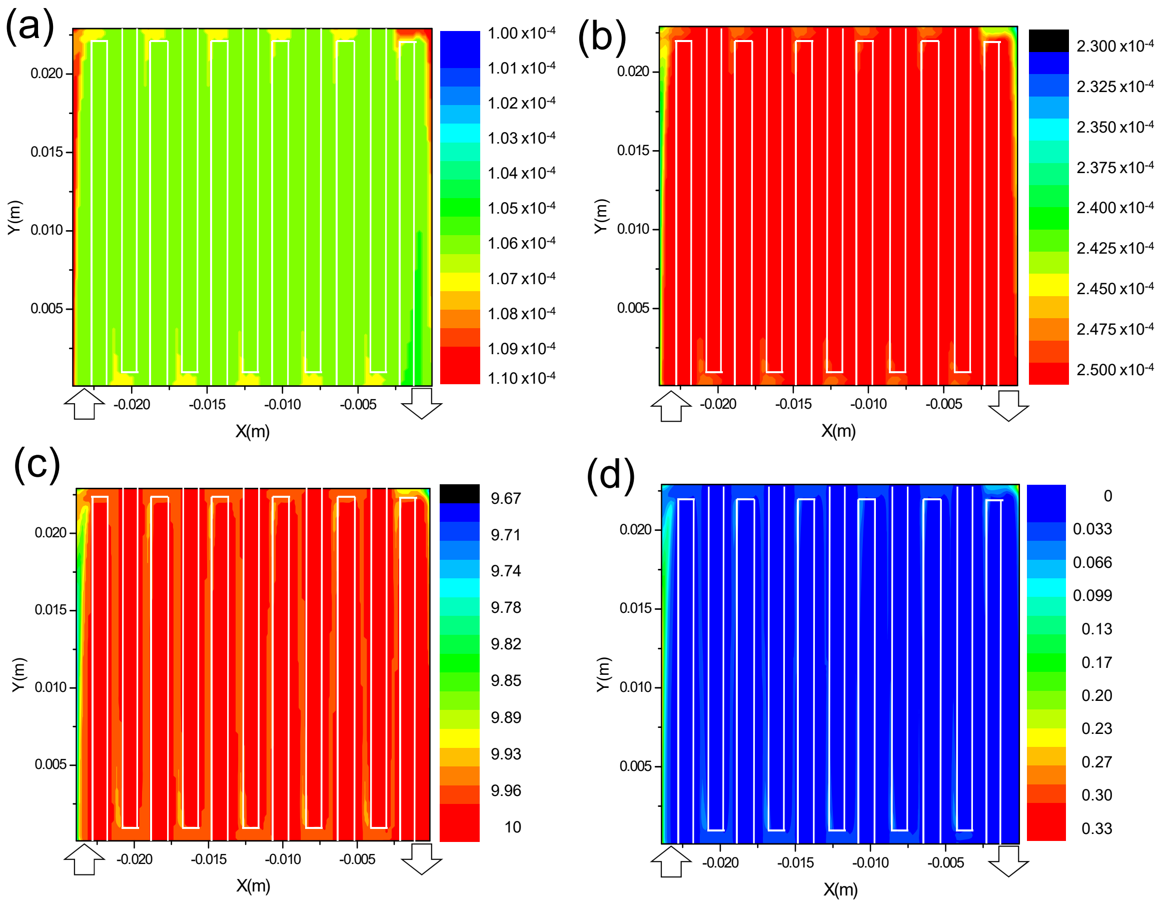

Figure S3. Simulated 2D concentration contour plots (in mole/m^3) of four active species: (a) holes, (b) electrons, (c) VO^{2+}, and (d) VO_2^+, along the midplane of the photoelectrode under a photocurrent of 0.5 mA and electrolyte flow rate of 2.0 ml/s.

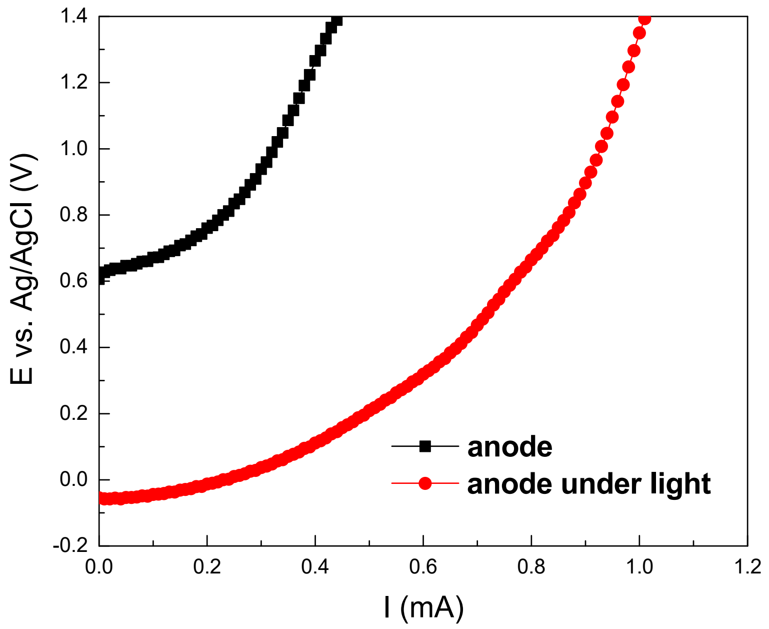

Figure S4. Photoelectrochemical performance of the photoanode half-cell. The test was performed in a H-shape cell where the TiO_2 photoelectrode served as the working electrode in the VO^{2+} (0.01 M) anolyte chamber, a Pt cathode as the counter electrode in the V^{3+} catholyte chamber, and a Ag/AgCl reference electrode placed in the anolyte chamber. The anolyte and catholyte were separated by a Nafion 117 membrane.

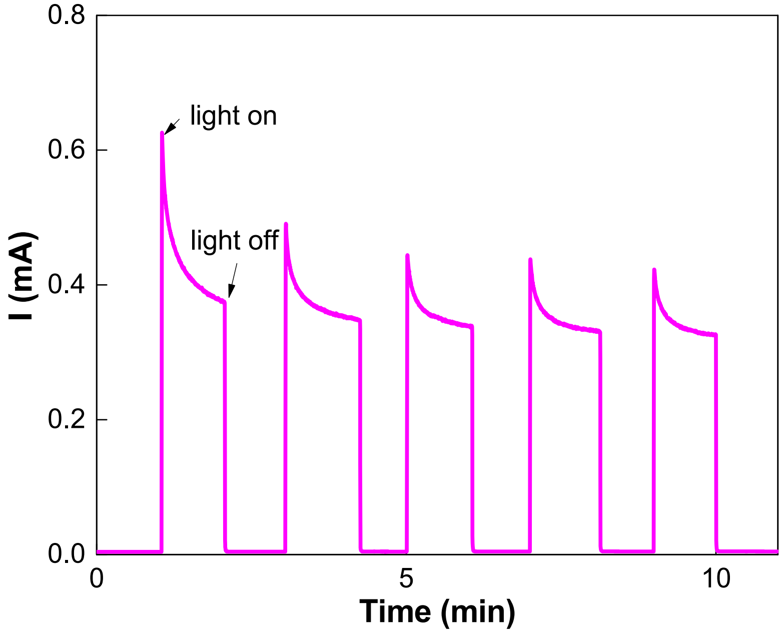

Figure S5. Photoelectrochemical performance under light on and off cycles. The test was performed in a H-shape cell where the TiO_2 photoelectrode served as the working electrode in the VO^{2+} (0.01 M) anolyte chamber and a Pt cathode as the counter electrode in the V^{3+} catholyte chamber. The anolyte and catholyte were separated by a Nafion 117 membrane.

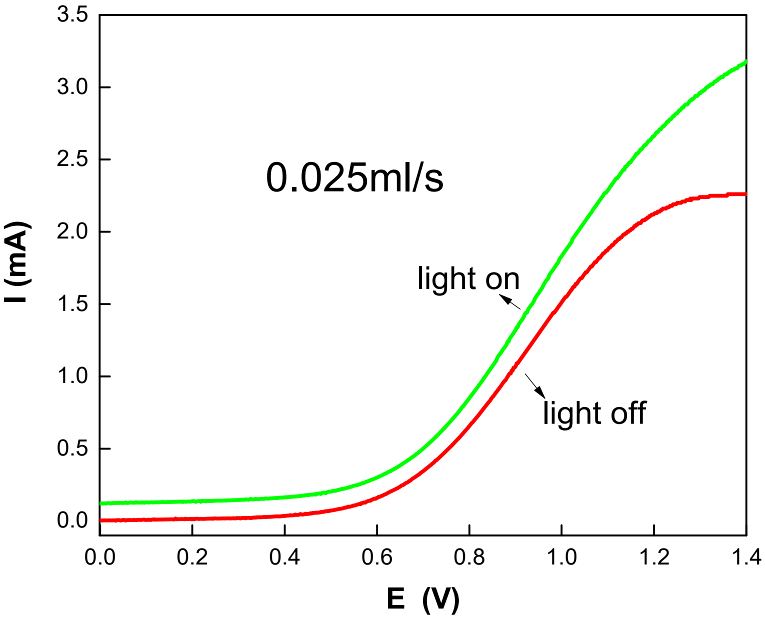

Figure S6. Linear sweep voltammograms of the all-V Continuous-flow PESC with light on and off. The results were obtained under flow rates of both anolyte and catholyte (both 0.01 M) at 0.025ml/s. The scanning rate is 20mV/s.

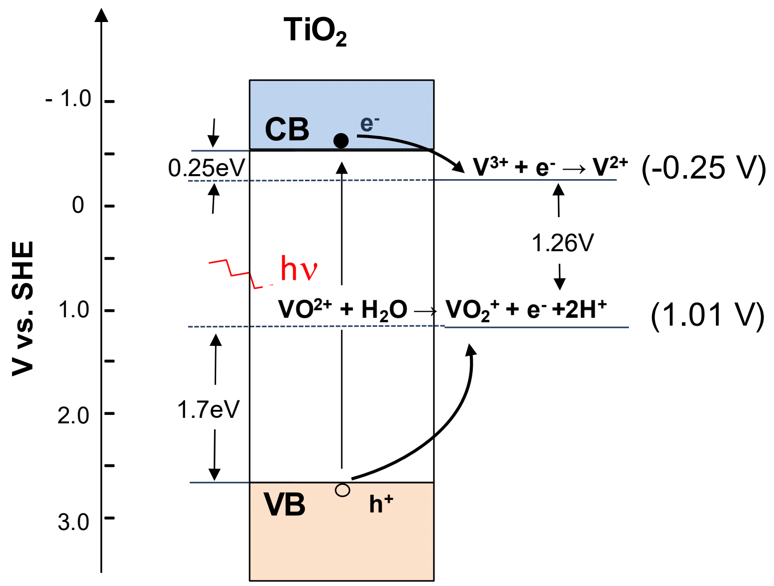

Figure S7. Energy diagram showing the band positions of TiO_2 and the electrochemical potentials of vanadium redox couples.

References:

1 Andrade, L., Sousa, J., Aguilar Ribeiro, H. & Mendes, A. Phenomenological modeling of dye-sensitized solar cells under transient conditions. *Solar Energy* **85**, 781-793, (2011).

2 Andrade, L., Lopes, T., Ribeiro, H. A. & Mendes, A. Transient phenomenological modeling of photoelectrochemical cells for water splitting – Application to undoped hematite electrodes. *International Journal of Hydrogen Energy* **36**, 175-188, (2011).

3 Khazaeli, A., Vatani, A., Tahouni, N. & Panjeshahi, M. H. Numerical investigation and thermodynamic analysis of the effect of electrolyte flow rate on performance of all vanadium redox flow batteries. *Journal of Power Sources* **293**, 599-612, (2015).

4 You, D., Zhang, H. & Chen, J. A simple model for the vanadium redox battery. *Electrochimica Acta* **54**, 6827-6836, (2009).

5 Al-Fetlawi, H., Shah, A. A. & Walsh, F. C. Modelling the effects of oxygen evolution in the all-vanadium redox flow battery. *Electrochimica Acta* **55**, 3192-3205, (2010).

6 Sun, C., Chen, J., Zhang, H., Han, X. & Luo, Q. Investigations on transfer of water and vanadium ions across Nafion membrane in an operating vanadium redox flow battery. *Journal of Power Sources* **195**, 890-897, (2010).

7 Boettcher, P. A., Agar, E., Dennison, C. R. & Kumbur, E. C. Modeling of Ion Crossover in Vanadium Redox Flow Batteries: A Computationally-Efficient Lumped Parameter Approach for Extended Cycling. *Journal of the Electrochemical Society* **163**, A5244-A5252, (2016).

8 Chen, C. L., Yeoh, H. K. & Chakrabarti, M. H. An enhancement to Vynnycky's model for the all-vanadium redox flow battery. *Electrochimica Acta* **120**, 167-179, (2014).
